# Differing Effects of Implementation Leadership Characteristics on Nurses’ Use of mHealth Technologies in Clinical Practice: Cross-Sectional Survey Study

**DOI:** 10.2196/44435

**Published:** 2023-08-25

**Authors:** Charlene Esteban Ronquillo, V Susan Dahinten, Vicky Bungay, Leanne M Currie

**Affiliations:** 1 School of Nursing, The University of British Columbia Okanagan Kelowna, BC Canada; 2 School of Nursing, The University of British Columbia Vancouver Vancouver, BC Canada

**Keywords:** mobile health, mHealth applications, nursing, leadership, implementation science, nursing informatics

## Abstract

**Background:**

Leadership has been consistently identified as an important factor in shaping the uptake and use of mobile health (mHealth) technologies in nursing; however, the nature and scope of leadership remain poorly delineated. This lack of detail about what leadership entails limits the practical actions that can be taken by leaders to optimize the implementation and use of mHealth technologies among nurses working clinically.

**Objective:**

This study aimed to examine the effects of first-level leaders’ implementation leadership characteristics on nurses’ intention to use and actual use of mHealth technologies in practice while controlling for nurses’ individual characteristics and the voluntariness of use, perceived usefulness, and perceived ease of use of mHealth technologies.

**Methods:**

A cross-sectional exploratory correlational survey study of registered nurses in Canada (n=288) was conducted between January 1, 2018, and June 30, 2018. Nurses were eligible to participate if they provided direct care in any setting and used employer-provided mHealth technologies in clinical practice. Hierarchical multiple regression analyses were conducted for the 2 outcome variables: intention to use and actual use.

**Results:**

The implementation leadership characteristics of first-level leaders influenced nurses’ intention to use and actual use of mHealth technologies, with 2 moderating effects found. The final model for intention to use included the interaction term for implementation leadership characteristics and education, explaining 47% of the variance in nurses’ intention to use mHealth in clinical practice (*F*_10,228_=20.14; *P*<.001). An examination of interaction plots found that implementation leadership characteristics had a greater influence on the intention to use mHealth technologies among nurses with a registered nurse diploma or a bachelor of nursing degree than among nurses with a graduate degree or other advanced education. For actual use, implementation leadership characteristics had a significant influence on the actual use of mHealth over and above the control variables (nurses’ demographic characteristics, previous experience with mHealth, and voluntariness) and other known predictors (perceived usefulness and perceived ease of use) in the model without the implementation leadership × age interaction term (β=.22; *P*=.001) and in the final model that included the implementation leadership × age interaction term (β=−.53; *P*=.03). The final model explained 40% of the variance in nurses’ actual use of mHealth in their work (*F*_10,228_=15.18; *P*<.001). An examination of interaction plots found that, for older nurses, implementation leadership characteristics had less of an influence on their actual use of mHealth technologies.

**Conclusions:**

Leaders responsible for the implementation of mHealth technologies need to assess and consider their implementation leadership behaviors because these play a role in influencing nurses’ use of mHealth technologies. The education level and age of nurses may be important factors to consider because different groups may require different approaches to optimize their use of mHealth technologies in clinical practice.

## Introduction

### Background

The use of mobile health (mHealth) technologies—mobile computing and information and communication technologies to support health care systems, health service delivery, and the achievement of specific health objectives [1]—continues to be recognized, with these applications being used as innovative tools within health systems. As the largest group of health professionals [2], nurses are the largest group targeted as end users of mHealth applications. The size of the nursing workforce highlights the role of nurses—whether willingly or otherwise—as direct shapers of the potential for success or failure of the deployment and use of mHealth applications in health systems. Recognizing the central role of nurses in determining the success or failure of mHealth applications, coupled with the attractive vision of materializing transformative improvements in health outcomes by equipping the vast nursing workforce with mHealth applications, there are ongoing efforts to better understand how mHealth applications can be used to support nurses’ work.

The dominant tools used to understand nurses’ use of mHealth applications have laid important foundations and provided crucial insights [3-5], although there are notable limitations to these approaches. The understanding of nurses’ adoption and use of mHealth applications is largely informed by models and frameworks of technology acceptance from the field of information science that have been used to understand the acceptance of various types of technologies among different end-user groups. Technology acceptance models approach the understanding of nurses’ use of mHealth applications through the examination of individual-level factors (eg, computer self-efficacy and perceived pressure to use the technology), with the premise that individuals’ attitudes toward technology ultimately shape use behaviors. Despite technology acceptance models growing in complexity through the incorporation of more variables that are thought to shape technology use behaviors (eg, the development of the Unified Theory of Acceptance and Use of Technology [UTAUT] [6-8] and the various iterations of the Technology Acceptance Model 3 [TAM3] [9]), there remains a limited ability to consider the role of structural factors in shaping technology use intentions and behaviors.

Leadership is a fundamental aspect of the nursing profession and an important structural factor that has been found to influence both nursing and patient outcomes [10-12]. The importance of leadership in nursing is further evidenced by position statements both nationally [13-15] and internationally [16-18]. The role of leadership is a commonality as a structural factor of leadership: as related to the use of mHealth applications and other health technologies by nurses [5,19-21], as related to the implementation of new innovations and evidence-based practice in nursing [22,23], and in implementation science models and frameworks [24-28]. Taken together, the consistent message is that *leadership is important* in shaping the use of mHealth applications, nursing practice, and implementation outcomes. However, a challenge in enabling effective leadership to support mHealth use is a lack of specificity as to what leadership entails and distinguishing the nature of leadership that is being referred to (eg, the characteristics of leaders, leadership behaviors, and supports provided by leaders). In the context of nurses’ use of mHealth applications, there remains limited consideration of the structural factor of leadership in influencing nurses’ mHealth use behaviors.

### Implementation Leadership and First-Level Leaders

Implementation leadership is specifically concerned with the leadership behaviors of local-level leaders or *first-level* leaders because they are well positioned to facilitate the implementation of innovations [29] and are deemed critical to organizational effectiveness [30]. First-level leaders are described as those who supervise individuals providing direct services [29]. In nursing, first-level leaders would be individuals who oversee nurses providing direct patient care (eg, educators, charge nurses, and ward managers), thus having influence and decision-making responsibilities at the local unit or department level. In nursing, first-level leadership is commonly referred to as *unit-level* leadership, and there is support for the importance of these leaders in influencing the implementation and uptake of practice changes and other innovations among nurses [31-33].

### Objectives

The objective of this study was to investigate the influence of leadership characteristics and behaviors specific to the implementation process (ie, implementation leadership characteristics) on Canadian nurses’ use of mHealth applications in clinical practice while controlling for the known predictors of the use of mHealth applications and technology. We sought to address this objective by answering the following 3 research questions (RQs):

- RQ1: What is the relationship between (1) implementation leadership characteristics and (2) nurses’ intention to use and (3) actual use of mHealth applications, after controlling for (4) perceived usefulness and perceived ease of use, (5) nurses’ previous experience with mobile technology and voluntariness of use, and (6) nurses’ demographic characteristics?
- RQ2: Do nurses’ (1) demographic characteristics moderate the relationship between (2) implementation leadership characteristics and (3) nurses’ intention to use and (4) actual use of mHealth applications?
- RQ3: Do nurses’ (1) voluntariness of use moderate the relationship between (2) implementation leadership characteristics and (3) nurses’ intention to use and (4) actual use of mHealth applications?

## Methods

### The Nurse Leadership for Implementing Technologies-Mobile Health Model

**Figure 1:**
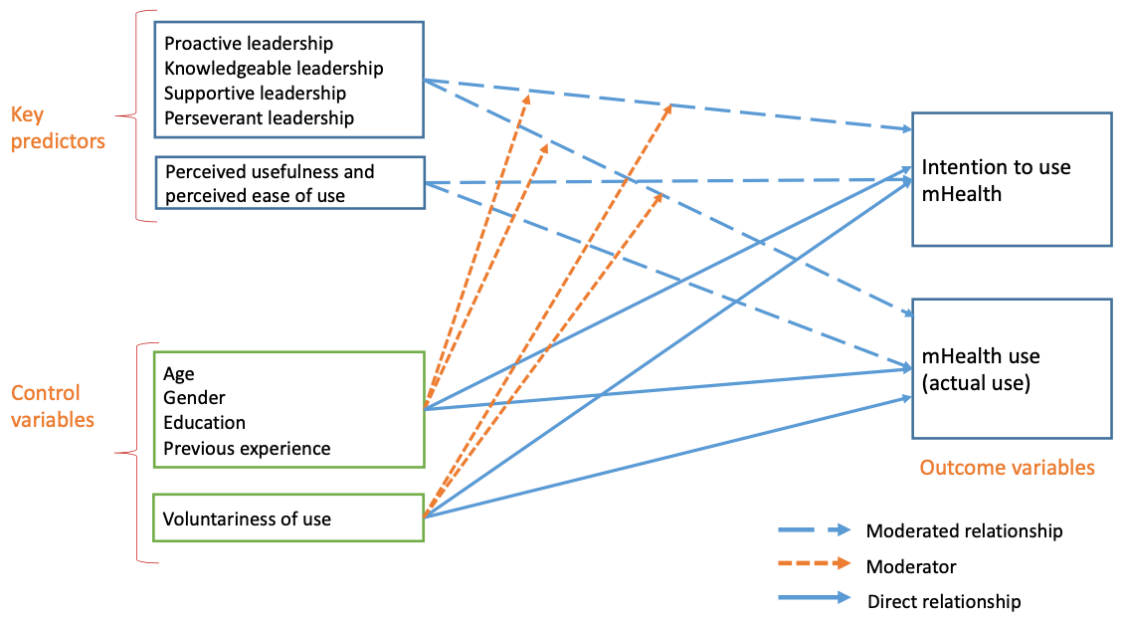
The Nurse Leadership for Implementing Technologies-Mobile Health model. mHealth: mobile health.

We developed the Nurse Leadership for Implementing Technologies-Mobile Health (Nurse LEAD-IT mHealth) conceptual model (Figure 1) to guide the conduct of the study. There are several well-established factors that have been found to influence the use of mHealth technologies, health information technology (HIT), and research among nurses and other health care professionals [3-6,16,17,33-35]. These characteristics are drawn from popular technology acceptance models and nurses’ research use literature and include individuals’ perceived usefulness and perceived ease of use of a technology, voluntariness of use, previous experience with technology, and demographic characteristics (age, gender, and education). The initial development of the Nurse LEAD-IT mHealth model has been described in detail elsewhere, including a more fulsome discussion of the definition and boundaries of leadership considered [34]; the model was further refined as the study progressed.

Understanding the unique role of leadership in influencing nurses’ use of mHealth technologies requires an approach that leverages what is currently known about the factors that influence nurses’ use of mHealth applications, HIT, and research as well as what is known about the role of leadership and how these influence the use of mHealth applications in nursing. As such, the NURSE LEAD-IT mHealth model integrates the known individual-level predictors of technology acceptance and use from the TAM3 [9], individual-level predictors of nurses’ uptake and use of evidence-based practice [35-37], and the structural factor of implementation leadership characteristics to consider the importance of leadership in nursing. In the model (Figure 1), implementation leadership characteristics as well as the perceived usefulness and perceived ease of use of mHealth technologies are suggested to be associated with the intention to use and actual use of mHealth applications. Nurses’ voluntariness of use and individual characteristics, which include demographics (age, gender, and education) and characteristics related to technology (previous experience with technology), are also considered in this model. Age, gender, education, and previous experience are posited to have direct effects on nurses’ intention to use and actual use of mHealth applications. In addition, age, gender, education, previous experience, and voluntariness are suggested to exert moderating effects on the associations between key predictor variables (implementation leadership characteristics, perceived usefulness, and perceived ease of use) and the intention to use and actual use of mHealth applications. The process of development and an in-depth discussion of the theoretical foundations of the Nurse LEAD-IT mHealth model are described elsewhere [34,38], and this study represents the first instance of its operationalization and use.

### Study Design

This was a cross-sectional exploratory correlational study. Study data were collected via an open web-based survey that was advertised and distributed to registered nurses (RNs) across Canada. In the following subsections, the web-based survey procedures are reported in accordance with the CHERRIES (Checklist for Reporting Results of Internet E-Surveys) [39].

### Participant Inclusion Criteria

Study participants were RNs in Canada who had workplace-provided access to mHealth applications as a tool to support the delivery of direct patient care. The targeted sample for this study met the following inclusion criteria: they (1) held RN licensure in Canada, (2) provided direct patient care in any setting, (3) had access to employer-provided mHealth applications for use in the provision of direct patient care, and (4) spoke English. Participants were restricted to RNs (ie, we excluded licensed practical nurses, registered psychiatric nurses, and nurse practitioners owing to differences in the scopes of practice and autonomy regarding mHealth tools used in practice that may exist among different types of nurses).

### Ethics Approval

Ethics approval was obtained from the University of British Columbia research ethics board (number: H17-02831).

### Recruitment and Informed Consent

Given the exploratory nature of the study, both convenience sampling and snowball sampling were used. The nature of nurses’ use of mHealth applications in Canada remains largely unknown, which did not allow for a more focused sampling strategy. More specifically, the dispersed nature of nurses’ use of mHealth applications in the Canadian context required a broad reach that did not prematurely narrow down to a specific type of clinical service, setting, or type of mHealth application.

The study was advertised via the researchers’ contacts in research and higher education organizations, as well as health and nursing informatics professional groups. The use of social media also constituted a major aspect of recruitment because nurses have been found to have high rates of social media use [40,41]. Survey advertisements were posted on publicly available professional nursing forums and groups on Facebook, Twitter, and LinkedIn. A dedicated web page and Facebook page with survey information were created. Paid advertisements were used on Facebook and Twitter to increase the visibility of the study pages. Information about the web-based survey was also distributed via specialty discussion lists such as JISCMail, which delivers messages to mailing list subscribers via email. Participant recruitment was also conducted via provincial RN regulatory bodies in Canada; however, recruitment in some provinces was not possible owing to regulatory or financial constraints (eg, Quebec was excluded because French is the primary language spoken in the province according to the regulatory body statements, and our study was conducted only in English).

Interested potential participants were directed to a landing page with information about the aims of the study, the survey structure, and the anticipated length of time it would take to complete the survey. Potential participants were required to answer screening questions to assess whether they met the eligibility criteria. Participants who met the eligibility criteria were redirected to the informed consent web page. After providing their consent by clicking *Start*, participants were able to begin the full survey, which included detailed instructions on how to complete the survey and the operational definitions of the terms used in the survey.

An incentive for participation was provided in the form of entry to a gift card draw for each week that the survey was open, an approach found to be successful in encouraging participation among nursing groups [42] and in increasing the odds of response [43]. Participants had the opportunity to enter prize draws for electronic gift certificates for the duration of the data collection period ($15 CAD weekly for each week the survey was open and $150 CAD at the end of the study). The prize incentive was of a small enough monetary value to ensure that escalating incentive amounts did not unduly influence responses or coerce participants [44]. Data were collected from January 1, 2018, to June 30, 2018.

### Data Collection

#### Web-Based Survey Development

We developed a web-based survey that consisted of existing scales, demographic questions, and researcher-developed questions using the tailored design method described by Dillman et al [45] and best practice recommendations on developing and conducting web-based surveys [46]. The 80-item web-based survey included five sections that addressed (1) the nature of the use of mHealth applications in practice, (2) nurses’ perspectives regarding their use of mHealth applications at work, (3) the characteristics (eg, job title) of leaders responsible for implementing mHealth applications, (4) previous experience with mHealth applications and other mobile technologies, and (5) nurses’ individual characteristics. The survey was pretested by nurses who met the inclusion criteria for potential respondents but were not involved in any other aspect of the study as per guidance [45]. Pretesting focused on the clarity and readability of content, accessibility, presentation and aesthetics, respondent burden, and ease in using and navigating the web survey, as well as other web survey–related considerations [45,47]. Revisions that involved the correction of typos and grammatical errors were carried out to develop the final version of the study survey, which was then uploaded to the university-provided Qualtrics survey software (which stores data on Canadian servers) for web-based distribution.

#### Variables and Instruments

The survey included an adapted version of the system-use measure developed by Doll and Torkzadeh [48,49], the Implementation Leadership Scale (ILS) developed by Aarons et al [26], variables from the TAM3 [9] and UTAUT [8], nurse demographic characteristics, and researcher-developed questions on the nature of the use of mHealth applications in nursing (eg, the functions of mHealth applications that were used).

#### Outcome Variables

The outcome variables were nurses’ intention to use mHealth applications and their actual use of mHealth applications. Intention to use refers to nurses’ plan to use mHealth applications as part of their clinical practice. The intention to use a technology is often considered a precursor or proxy for actual technology use behaviors; it was considered as the latter in this study. The measure for intention to use comprised 3 items adapted from the TAM3 developed by Venkatesh and Bala [9]. To mitigate the limitations of using only intention to use as the measure of nurses’ use of mHealth applications, actual use was also captured. We used the measure of system use developed by Doll and Torkzadeh [48] as adapted by Maillet [49], which has been validated in the context of the Canadian health care system (Cronbach α=.93).

#### Predictor Variables

The key predictor variables in this study were implementation leadership characteristics as well as perceived usefulness and perceived ease of use of technology. Implementation leadership characteristics were measured using the staff version of the ILS [26]. The ILS asks respondents to reflect on the specific leadership behaviors of the *first-level* leader in charge of the implementation of mHealth applications, recognizing their key positioning to facilitate implementation [26,29]. To identify first-level leaders, participants were asked to identify the formal title of the person responsible for introducing mHealth and the formal title of the person responsible for maintaining ongoing mHealth use to support nursing practice (unit or department manager, charge nurse, clinical nurse educator, resource person outside of the unit or department, or other). Perceived usefulness refers to nurses’ perceptions of how useful mHealth applications are in their work. The measure of perceived usefulness was adapted from the TAM3 developed by Venkatesh and Bala [9]. Perceived ease of use refers to one’s perception of how easy it is to use mHealth applications. The measure of perceived ease of use was adapted from the TAM3 developed by Venkatesh and Bala [9] and comprises a subset of 4 items from the early studies on the user acceptance of computer technology conducted by Davis et al [7].

#### Control Variables

Control variables included voluntariness (a technology characteristic), previous experience with technology (individual characteristic related to technology), and nurse demographic characteristics (age, gender, and education). Voluntariness refers to the degree to which the use of mHealth applications is either a mandatory component or a voluntary component of nurses’ jobs. Three items, drawn from the study by Moore and Benbasat [50], were used to measure voluntariness. Previous experience was conceptualized in this study similar to previous studies, with experience representing the passage of time from the initial use of the technology up to the present [8,51]. Nurse demographic characteristics included age, gender, and education, reflecting individual characteristics identified in both the technology use literature and nurses’ research use literature. Age in years was calculated from the participant’s report of their year and month of birth. Although studies on nurses’ use of research have found no association between age and nurses’ research use [35,36], age has been identified in the technology use literature as influencing individuals’ perceived ease of use, perceived usefulness, and attitudes toward technology, with older participants expressing less positive attitudes toward technology [8,9].

Information on gender was collected by asking respondents to identify as men, women, prefer not to say, or other. Previous research has found that gender roles and norms influence attitudes toward, and actual use of, technology [8,52]. Information on education was collected by asking participants to indicate the highest type of nursing degree that they had completed (RN diploma, bachelor of nursing degree, master of nursing degree, or PhD). For analyses, nursing education was dichotomized into two groups: (1) RN diploma or bachelor of nursing degree group and (2) nursing graduate degree or other advanced education group. This grouping was based on findings from the research use literature where having a graduate degree was associated with increased research use compared with having a diploma or a bachelor of nursing degree, with no differences found when comparing research use between nurses with bachelor of nursing degrees and those with diplomas [36].

All instruments were psychometrically evaluated. Principal component analyses for all scales produced component solutions consistent with previous studies [6,7,9,52-55], with the exception of the system-use measure developed by Doll and Torkzadeh [48], which extracted 2 components that explained 67.74% of the variance. These findings do not reflect the 5-dimension structure of actual use as proposed in the original scale developed by Doll and Torkzadeh [48]. However, the 2-component solution does reflect the findings of the scale as adapted and used among nurses by Maillet [49]. A summary of the component structure statistics for all scales used in each group of variables (outcome variables, predictor variables, and control variables) is outlined in Multimedia Appendix 1 [6-9,48,49,51-55].

### Statistical Analyses

#### Power

An a priori sample size calculation to detect a small effect and achieve statistical power of 0.8, with α<.05 for a hierarchical multiple regression, was computed; a minimum of 177 participants was required. A small effect size was used as a conservative estimate, given the lack of information on the potential effects of leadership behaviors on the uptake and use of mHealth applications. Responses from 288 participants were used in all regression analyses, which ensured sufficient statistical power.

#### Data Screening and Preparation

Data were extracted from the web-based Qualtrics software into a password-protected SPSS (version 26.0; IBM Corp) database, and the raw data were screened for missing, incorrect, and questionable response patterns as well as data entry errors [56]. A review of the responses to the question asking about the type of nursing registration revealed that 35 (9%) of the 388 participants did not meet the inclusion criteria because they were registered practical nurses (n=7, 20%), nurse practitioners (n=27, 77%), or registered midwives (n=1, 3%); these responses were excluded from analyses. Other cases were removed owing to missing ILS scores (65/388, 16.7%). Thus, of the initial 388 participants, after removal of these 100 (25.8%) cases, 288 (74.2%) remained for analysis. Descriptive statistics (frequencies, percentages, means, SDs, and ranges) were obtained for each study variable and used to assess whether the data met the assumptions required to perform hierarchical multiple regression analyses [57,58]. Upon the completion of the data screening steps, all assumptions to conduct hierarchical multiple regression analyses were deemed to have been met.

#### Hierarchical Multiple Regression and Moderation Analyses

Hierarchical multiple regression was used as the main method of data analysis. Diagnostics of the *intention to use* and *actual use* regression models were conducted to assess model assumptions; all assumptions were met. Diagnostic assessments included an examination of Q-Q plots and residuals scatterplots to inspect the normality of residuals and to visually identify potential outliers; standardized residuals, leverage values, Cook distance, and Mahalanobis distance to assess model fit with the data and identify potentially influential cases; Durbin-Watson statistic to assess independent errors (ie, independence of residual terms for any 2 observations); and intraclass correlations to assess the independence of observations, multicollinearity among independent variables (indicated by multicollinearity indices [variance inflation factor {<10} and tolerance {>0.1}]), homoscedasticity (by examining the scatterplot of the standardized errors [y-axis] against the standardized predicted Y [x-axis]), and skew, kurtosis, and normal distribution of residuals, as well as the absence of extreme multicollinearity in the model variables [56,59,60].

The creation of the regression model and the sequence in which variables were entered into the model were theoretically justified and as detailed in the development of the conceptual model [34] and guidance on the order of variable entry when conducting a hierarchical multiple regression analysis [56,59]. Known predictors were entered first, followed by the key predictors of interest. Separate sets of models were run for each of the 2 outcome variables: intention to use mHealth applications and actual use of mHealth applications. This order of model entry aimed to examine the unique effect of implementation leadership characteristics over and above the effect of control variables, perceived usefulness, and perceived ease of use (RQ1) and whether there were significant moderating effects of nurse demographic variables (RQ2) and voluntariness (RQ3) on implementation leadership.

To test for moderating effects, an interaction term was produced for each interaction of interest, which is the product of the proposed moderator variable and the key predictor variable it is thought to influence [61]. Six interaction terms were computed and used in the regression analyses for each of the 2 outcome variables: implementation leadership × age, implementation leadership × gender, implementation leadership × education, implementation leadership × previous experience with work mHealth applications, implementation leadership × previous experience with nonwork mobile technology use, and implementation leadership × voluntariness. Each interaction term was tested independently in the final model for each outcome variable. Nonsignificant interaction terms were dropped in the final models. As no differences in outcomes were found with comparisons of centered outcome variables (creation of a new variable by subtracting the variable mean so that the new mean is 0) and noncentered outcome variables (preserving the original variable values; the mean will not be 0), this study used noncentered outcome variables to preserve the original scale of the variable, as per the recommendations by Aiken et al [61].

Local effect sizes resulting from the addition of variables to the regression models were calculated for significant variables. A variation of Cohen *f*^2^ as described by Selya et al [62] was calculated using the web-based Soper effect size calculator for hierarchical multiple regression analysis [63], which provides a measure of the effect size of the addition of variables to the regression models [64]. Effect sizes are reported as per guidance by the American Statistical Association [65] and recommendations by various authors [65-67]. Cohen *f*^2^ is a standardized measure of 1 variable’s local effect size in the context of a multivariate regression model (ie, the unique proportion of the variance accounted for by the variable of interest) [62,64].

## Results

### Participant Demographics

There were 388 responses to the survey. After removing cases that did not meet the inclusion criteria (35/388, 9%) or were missing ILS responses (65/388, 16.7%), the final sample out of 388 participants consisted of 288 (74.2%) RNs. Descriptive summaries of participant demographics are outlined in Tables 1 and 2. The proportion of men in the sample (16/288, 5.6%) reflects the proportion of men RNs found in the Canadian RN population in 2019 of 7.5% (range 4.6% [in Prince Edward Island] to 11.5% [in Quebec]) [68]. Most of the respondents were RNs working in large urban settings (163/288, 56.6%) and primarily in hospital (110/288, 38.2%) or community health (117/288, 40.6%) settings.

Although participants were asked to indicate their primary nursing job, they were permitted to provide multiple responses, which resulted in 470 total responses (Table 3). Six practice specialties made up the most commonly reported specialty areas of practice by respondents (331/470, 70.4%). In descending order of frequency, respondents practiced in community or public health, medical, geriatrics or care of older people, emergency care, home care, and surgical nursing, followed by smaller numbers reported for the remaining practice specialty areas.

**Table 1:** Participant demographic characteristics (n=288).

Characteristics			Values
Age (years), mean (SD)			41.6 (11.9)
**Age group (years), n (%)**			
	≤29	58 (20.1)	
	30-39	89 (30.9)	
	40-49	60 (20.8)	
	50-59	53 (18.4)	
	≥60	25 (8.7)	
**Gender, n (%)**			
	Women	272 (94.4)	
	Men	16 (5.6)	
**Highest educational qualification in nursing, n (%)**			
	RN^a^ diploma	64 (22.2)	
	Bachelor of nursing degree	195 (67.7)	
	Master of nursing degree	21 (7.3)	
	PhD (nursing)	1 (0.3)	
	Other (eg, advanced practice and specialty diplomas)	7 (2.4)	
Years since first obtaining RN license, mean (SD)			16.9 (12.6)

^a^RN: registered nurse.

**Table 2:** Participant employment characteristics (n=288).

Characteristic		Participants, n (%)^a^
**Canadian province^b^ of employment**		
	British Columbia	33 (11.5)
	Alberta	63 (21.9)
	Saskatchewan	128 (44.4)
	Manitoba	13 (4.5)
	Ontario	5 (1.7)
	New Brunswick	21 (7.3)
	Newfoundland and Labrador	24 (8.3)
**Type of population setting^c^**		
	Large urban population center	163 (56.6)
	Medium population center	40 (13.9)
	Small population center	36 (12.5)
	Rural area	48 (16.7)
**Organization type**		
	Hospital	110 (38.2)
	Community health	117 (40.6)
	Nursing home or other long-term care facility	31 (10.8)
	Other	27 (9.4)

^a^The sums of the characteristics do not equal 288 owing to missing responses and the data are not reported here, as per the conventions of reporting missing data.

^b^The numbers of participant respondents per province do not reflect the distribution of nurses across Canada’s 10 provinces and 3 territories.

^c^Large urban population center: >100,000 people (high population density), medium population center: between 30,000 and 99,999 people (high population density), small population center: between 1000 and 29,999 people (high population density), and rural area: all other areas outside of population centers (extracted from Population Centre and Rural Area Classification 2016 [69]).

**Table 3:** Respondents’ areas of practice (n=470^a^).

Practice specialty of primary nursing job	Respondents, n (%)
Community or public health	86 (18.3)
Medical	68 (14.5)
Geriatrics or care of older people	55 (11.7)
Emergency care	44 (9.4)
Home care	39 (8.3)
Surgical	39 (8.3)
Critical care	27 (5.7)
Maternal	21 (4.5)
End of life	21 (4.5)
Pediatrics	20 (4.3)
Psychiatry or mental health	18 (3.8)
Other	13 (2.8)
Clinical or health informatics	6 (1.3)
Occupational health	5 (1.1)
Primary care^b^	4 (0.9)
Administration^c^	3 (0.6)
Correctional	1 (0.2)

^a^Participants were asked to choose all that apply, resulting in a total frequency of 470.

^b^Primary care was a new category identified in the text responses to the “Other, please describe” option.

^c^Individuals who indicated administration also selected >1 practice specialty area and indicated their work setting to be in a primary care clinic.

### Descriptive Statistics

Descriptive statistics of the outcome variables (intention to use and actual use), key predictor variables (implementation leadership characteristics, perceived usefulness, and perceived ease of use), and control variables (voluntariness, previous experience with work mHealth applications, and nonwork mobile technology use) are reported in Table 4. Histograms for the variables perceived usefulness, perceived ease of use, previous experience with work mHealth applications, and voluntariness were skewed; therefore, medians are reported for these variables (unlike means, medians are less sensitive to skewed distributions [59]).

Overall, scores for the outcome variables indicated relatively high intention to use mHealth and moderately high actual use of mHealth among respondents (Table 4). For key predictor variables, the respondent scores indicated moderate perceptions of implementation leadership characteristics related to mHealth implementation. The median scores for the known predictors perceived usefulness and perceived ease of use suggested that respondents moderately perceived mHealth use at work to be useful and easy to use. There was a median of 48.95 months or 4.08 years of experience with work mHealth applications and an average of 162.48 (SD 79.63) months or 13.54 (SD 6.64) years of experience with nonwork mobile technology use. The median score for voluntariness suggested that nurses did not tend to perceive the use of mHealth in their work as voluntary.

The strengths of relationships among the major study variables were assessed via bivariate correlations using Pearson *r*, point-biserial correlations (*r_pb_*), and φ correlation analyses. None of the bivariate correlations among the independent variables or among the outcome variables were deemed highly correlated (ie, all *r* values were <0.8) [57,59,64]. A bivariate correlation matrix among all variables is outlined in Multimedia Appendix 2 [6,8,9,26,35,36,48-52,70,71]. On the basis of the results of the completed diagnostics, assumptions to perform regression analyses were met [59].

**Table 4:** Description of model variables.

Characteristics			Values, mean (SD)		Values, median		Values, range
**Outcome variables**							
	Intention to use	6.01 (1.13)		N/A^a^		2.00-7.00	
	Actual use	37.57 (12.66)		N/A		14.00-70.00	
**Key predictor variables**							
	Implementation leadership characteristics	2.13 (1.05)		N/A		0.00-4.00	
	Perceived usefulness	N/A		6.00		1.00-7.00	
	Perceived ease of use	N/A		5.25		1.00-7.00	
**Control variables^b^**							
	Previous experience with work mHealth^c^ applications (months)	N/A		48.95		0.36-339.34	
	Previous experience with nonwork mobile technology use (months)	162.48 (79.63)		N/A		0.36-342.46	
	Voluntariness	N/A		2.33		1.00-7.00	

^a^N/A: not applicable.

^b^The control variables age, gender, and education were described here.

^c^mHealth: mobile health.

### Hierarchical Regression Findings

#### Intention to Use mHealth Applications

Regression results for the final models predicting intention to use mHealth applications are reported in Table 5.

Model 4 shows that implementation leadership characteristics were not found to have a significant primary effect on nurses’ intention to use mHealth technologies (*P*=.12) after controlling for perceived usefulness, perceived ease of use (known key predictors), voluntariness of use (control variable), nurses’ demographic characteristics, and previous experience with mHealth applications and other mobile technologies (control variables). Model 5, which included the interaction term implementation leadership × education, explained 47% of the variance in nurses’ intention to use mHealth applications in their clinical practice (*F*_10,228_=20.14; *P*<.001). The effect size attributable to the addition of implementation leadership characteristics to model 4 is Cohen *f*^2^=0.01, and the effect size attributable to the addition of the implementation leadership × education interaction term to model 5 is Cohen *f*^2^=0.02; both are considered small effect sizes [72].

In the intention to use mHealth applications models, perceived usefulness (β=.45; *P*<.001) and perceived ease of use (β=.34; *P*<.001) were found to be the strongest predictors of nurses’ intention to use mHealth applications (*sr_i_*^2^=0.41). The addition of perceived usefulness and perceived ease of use accounted for 41% of the variance. A small to moderate effect size (Cohen *f*^2^=0.13) can be attributed to the addition of perceived usefulness and perceived ease of use [72].

Voluntariness was not found to moderate the relationship between implementation leadership characteristics and intention to use (*P*=.17). Voluntariness (β=−.21; *P*<.001) was found to be negatively associated with nurses’ intention to use mHealth applications in the final model. In other words, if nurses had an option regarding using mHealth applications in their work (ie, if use was voluntary), they had lower intention to use them.

**Figure 2:**
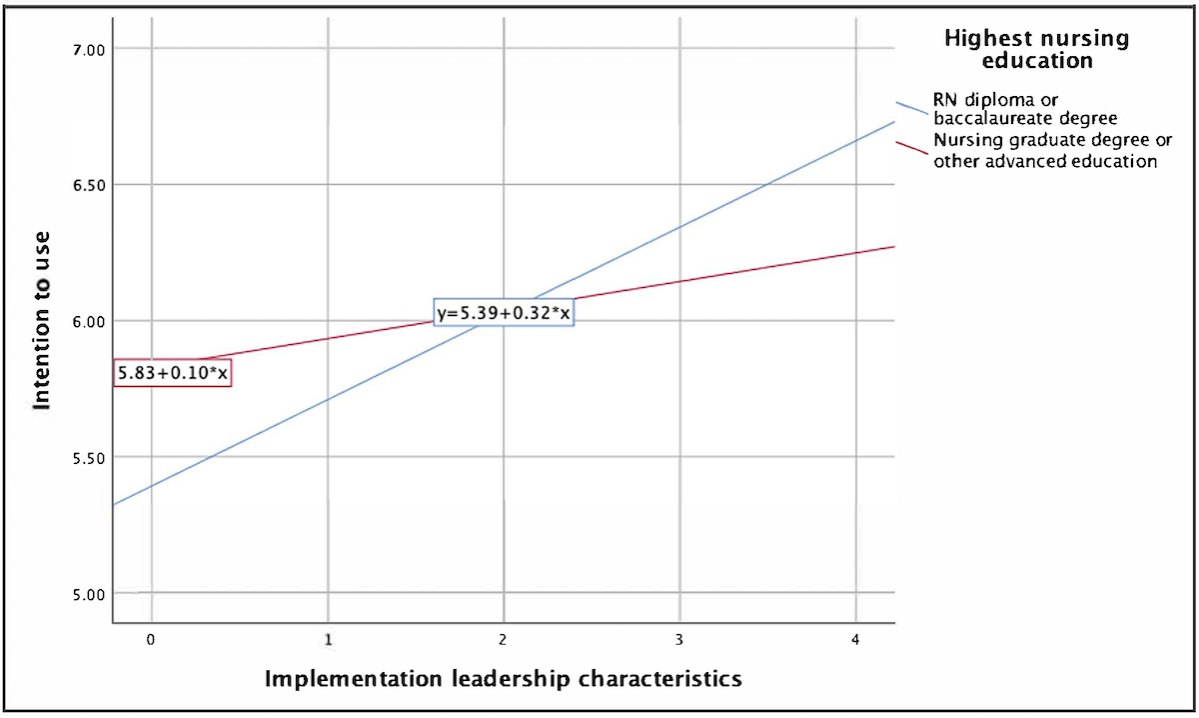
The moderating effect of education on the relationship between implementation leadership characteristics and nurses’ intention to use mobile health applications. RN: registered nurse.

The inclusion of the interaction term in the final model does not allow for the interpretation of the primary effects of implementation leadership on intention to use mHealth applications in the final model [56]. Only the implementation leadership × education interaction term [61] was statistically significant in the final model (β=−.21; *P*=.03) and was negatively associated with nurses’ intention to use mHealth applications. This significant negative β coefficient and the plotted interaction (Figure 2) suggest that education moderated the effect of implementation leadership characteristics on nurses’ intention to use mHealth applications.

The interaction plot in Figure 2 depicts simple regression lines that plot implementation leadership characteristics with intention to use mHealth applications for each education group (point-biserial correlation [*r_pb_*] between implementation leadership characteristics and nurse education; *r_pb_*=−0.16; *P*=.008). From this figure, it seems that perceptions of higher implementation leadership had a greater influence on the intention to use mHealth applications among nurses with an RN diploma or a bachelor of nursing degree than among those with a graduate degree or other advanced education. Moreover, lower levels of implementation leadership among nurses with graduate degrees were associated with higher intention to use mHealth applications compared with nurses with an RN diploma or a bachelor of nursing degree. However, at higher levels of implementation leadership, nurses with an RN diploma or bachelor of nursing degree showed higher levels of intention to use mHealth applications than those with a graduate degree or other advanced education, not controlling for other variables.

**Table 5:** Final regression models predicting intention to use mobile health (mHealth) applications (n=238).

Model and variable		B (SE; 95% CI)	β	*P* value	*R* ^2^		∆*R*^2^	
**Model 1**						0.004		0.004
	Gender^a^	−0.10 (0.32; −0.733 to 0.534)	−.02	.76				
	Education^b^	−0.12 (0.26; −0.620 to 0.386)	−.03	.65				
	Age	−0.00 (0.001; −0.015 to 0.014)	−.01	.93				
	Months of previous experience (work mHealth applications)	−0.00 (0.001; −0.003 to 0.002)	−.05	.53				
	Months of previous experience (nonwork mobile technology use)	0.00 (0.01; −0.002 to 0.002)	.02	.78				
**Model 2**						0.038		0.034
	Gender	−0.22 (0.32; −0.849 to 0.410)	−.05	.49				
	Education	0.01 (0.26; −0.495 to 0.512)	.00	.97				
	Age	−0.01 (0.01; −0.020 to 0.009)	−.06	.45				
	Months of previous experience (work mHealth applications)	−0.00 (0.001; −0.003 to 0.002)	−.03	.64				
	Months of previous experience (nonwork mobile technology use)	0.00 (0.001; −0.002 to 0.002)	−.00	.99				
	Voluntariness	−0.11 (0.04; −0.177 to −0.033)	−.20	.01				
**Model 3**						0.451		0.414
	Gender	0.10 (0.25; −0.380 to 0.585)	.02	.68				
	Education	0.15 (0.19; −0.229 to 0.536)	.04	.43				
	Age	0.00 (0.01; −0.011 to 0.012)	.01	.94				
	Months of previous experience (work mHealth applications)	−0.00 (0.001; −0.002 to 0.001)	−.03	.60				
	Months of previous experience (nonwork mobile technology use)	0.00 (0.001; −0.001 to 0.001)	.00	.99				
	Voluntariness	−0.09 (0.03; −0.145 to −0.035)	−.17	.001				
	Perceived usefulness	0.36 (0.05; 0.260 to 0.451)	.42	<.001				
	Perceived ease of use	0.29 (0.05; 0.185 to 0.386)	.33	<.001				
**Model 4**						0.457		0.006
	Gender	0.07 (0.25; −0.410 to 0.554)	.02	.77				
	Education	0.11 (0.20; −0.273 to 0.496)	.03	.57				
	Age	0.00 (0.01; −0.010 to 0.013)	.02	.77				
	Months of previous experience (work mHealth applications)	−0.00 (0.001; −0.003 to 0.001)	−.03	.56				
	Months of previous experience (nonwork mobile technology use)	0.00 (0.001; −0.001 to 0.002)	.01	.93				
	Voluntariness	−0.11 (0.03; −0.176 to −0.051)	−.21	<.001				
	Perceived usefulness	0.37 (0.05; 0.274 to 0.469)	.44	<.001				
	Perceived ease of use	0.30 (0.05; 0.201 to 0.406)	.35	<.001				
	Implementation leadership characteristics	−0.11 (0.07; −0.240 to 0.027)	−.10	.12				
**Model 5^c^**						0.469		0.012
	Gender	0.07 (0.24; −0.410 to 0.546)	.01	.78				
	Education	0.81 (0.36; 0.089 to 1.521)	.21	.03				
	Age	0.00 (0.01; −0.009 to 0.014)	.03	.64				
	Months of previous experience (work mHealth applications)	−0.00 (0.001; −0.003 to 0.001)	−.04	.43				
	Months of previous experience (nonwork mobile technology use)	0.00 (0.001; −0.001 to 0.002)	.01	.91				
	Voluntariness	−0.11 (0.03; −0.174 to −0.051)	−.21	<.001				
	Perceived usefulness	0.38 (0.05; 0.281 to 0.475)	.45	<.001				
	Perceived ease of use	0.29 (0.05; 0.193 to 0.396)	.34	<.001				
	Implementation leadership characteristics	−0.07 (0.07; −0.206 to 0.066)	−.07	.31				
	Implementation leadership × education	−0.43 (0.19; −0.811 to −0.054)	−.21	.03				

^a^0=man, 1=woman.

^b^0=registered nurse diploma or bachelor of nursing degree, 1=nursing graduate degree or other advanced education.

^c^*F*_10,228_=20.14; *P*<.001.

#### Actual Use of mHealth Applications

Regression results for the final models predicting actual use of mHealth applications are reported in Table 6. Implementation leadership characteristics were found to have a significant influence on actual use of mHealth applications over and above control variables and other known predictors in models 4 (β=.22; *P*=.001) and 5 (β=.63; *P*=.002). The final model explained 40% of the variance in nurses’ actual use of mHealth applications in their work (*F*_10,228_=15.18; *P*<.001). A Cohen *f*^2^ value of 0.20 was obtained, which represents a medium effect size attributable to the addition of the implementation leadership × age interaction term [72].

In model 2, gender was statistically significant (β=−.15; *P*=.02) along with voluntariness of use (β=−.38; *P*<.001). Voluntariness remained statistically significant in model 3 (β=−.35; *P*<.001), model 4 (β=−.25; *P*<.001), and model 5 (β=−.26; *P*<.001). Voluntariness was not found to moderate the relationship between implementation leadership characteristics and actual use. The addition of perceived usefulness was statistically significant in model 3 (β=.49; *P*<.001), and its addition to the model (along with perceived ease of use) had a moderate effect size (Cohen *f*^2^=0.33). Perceived usefulness was also significant in model 4 (β=.45; *P*<.001) and model 5 (β=.47; *P*<.001). Perceived usefulness was the strongest predictor of nurses’ actual use of mHealth applications (*sr_i_*^2^=0.21) in model 3.

**Figure 3:**
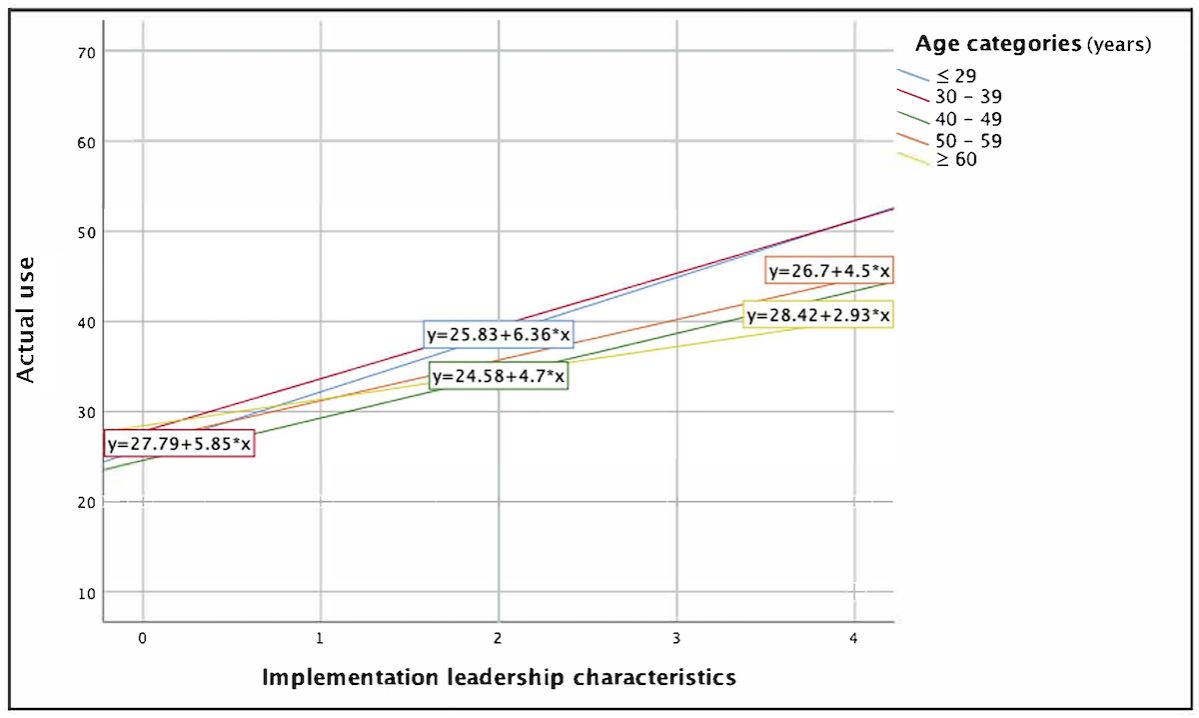
The moderating effect of age on the relationship between implementation leadership characteristics and nurses’ actual use of mobile health (mHealth) applications.

The inclusion of the interaction term in the final model does not allow for the interpretation of the primary effects of implementation leadership on actual use of mHealth applications in the final model [56]. Only the implementation leadership × age interaction term was found to be statistically significant (β=−.53; *P*=.03) and had the second largest β coefficient in the final model. This significant negative β coefficient and the plotted interaction in Figure 3 suggest that age moderates the effect of implementation leadership on nurses’ actual use of mHealth applications, with implementation leadership having a greater influence on the 3 youngest groups of nurses: those aged ≤29 years (*r*=0.49; *P*<.001), those aged 30 to 39 years (*r*=0.50; *P*<.001), and those aged 40 to 49 years (*r*=0.44; *P*<.001). A potential explanation of the nonsignificant effect of implementation leadership characteristics on the actual use of mHealth applications for nurses aged ≥60 years is that they constituted the smallest group in this sample, increasing the likelihood of a type II error.

**Table 6:** Final regression models predicting actual use of mobile health (mHealth) applications (n=238).

Model and variable			B (SE; 95% CI)		β		*P* value		*R* ^2^			∆*R*^2^	
**Model 1**										0.02			0.02
	Gender^a^	−0.38 (0.25; −0.870 to 0.116)		−.10		.13							
	Education^b^	−0.05 (0.20; −0.446 to 0.338)		−.02		.79							
	Age	−0.00 (0.01; −0.011 to 0.011)		.00		.99							
	Months of previous experience (work mHealth applications)	−0.00 (0.001; −0.003 to 0.001)		−.07		.32							
	Months of previous experience (nonwork mobile technology use)	−0.00 (0.001; −0.002 to 0.001)		−.01		.85							
**Model 2**										0.15			0.13
	Gender	−0.56 (0.24; −1.026 to −0.097)		−.15		.02							
	Education	0.14 (0.19; −0.232 to 0.510)		.05		.46							
	Age	−0.01 (0.01; −0.020 to 0.000)		−.10		.16							
	Months of previous experience (work mHealth applications)	−0.00 (0.001; −0.003 to 0.001)		−.05		.47							
	Months of previous experience (nonwork mobile technology use)	−0.010 (0.001; −0.002 to 0.001)		−.05		.41							
	Voluntariness	−0.16 (0.03; −0.2104to −0.108)		−.38		<.001							
**Model 3**										0.36			0.21
	Gender	−0.31 (0.21; −0.721 to 0.096)		−.08		.13							
	Education	0.22 (0.16; −0.109 to 0.539)		.07		.19							
	Age	−0.01 (0.01; −0.017 to 0.002)		−.10		.13							
	Months of previous experience (work mHealth applications)	−0.00 (0.001; −0.002 to 0.001)		−.05		.40							
	Months of previous experience (nonwork mobile technology use)	−0.00 (0.001; −0.002 to 0.001)		−.03		.62							
	Voluntariness	−0.15 (0.02; −0.194 to −0.101)		−.35		<.001							
	Perceived usefulness	0.32 (0.04; 0.241 to 0.404)		.49		<.001							
	Perceived ease of use	−0.03 (0.04; −0.118 to 0.052)		−.05		.45							
**Model 4**										0.39			0.03
	Gender	−0.26 (0.20; −0.661 to 0.142)		−.07		.20							
	Education	0.29 (0.16; −0.032 to 0.608)		.10		.08							
	Age	−0.01 (0.01; −0.019 to 0.000)		−.13		.05							
	Months of previous experience (work mHealth applications)	−0.00 (0.001; −0.002 to 0.001)		−.04		.47							
	Months of previous experience (nonwork mobile technology use)	−0.00 (0.001; −0.002 to 0.001)		−.04		.50							
	Voluntariness	−0.11 (0.03; −0.158 to −0.054)		−.25		<.001							
	Perceived usefulness	0.30 (0.04; 0.214 to 0.376)		.45		<.001							
	Perceived ease of use	−0.06 (0.04; −0.149 to 0.021)		−.09		.14							
	Implementation leadership characteristics	0.19 (0.06; 0.075 to 0.297)		.22		.001							
**Model 5^c^**										0.40			0.01
	Gender	−0.26 (0.20; −0.659 to 0.138)		−.07		.20							
	Education	0.28 (0.16; −0.041 to 0.594)		.09		.09							
	Age	−0.01 (0.01; −0.011 to 0.028)		−.11		.38							
	Months of previous experience (work mHealth applications)	−0.00 (0.001; −0.002 to 0.001)		−.04		.45							
	Months of previous experience (nonwork mobile technology use)	−0.00 (0.001; −0.002 to 0.001)		−.05		.41							
	Voluntariness	−0.11 (0.03; −0.163 to −0.059)		−.26		<.001							
	Perceived usefulness	0.31 (0.04; 0.225 to 0.388)		.47		<.001							
	Perceived ease of use	−0.07 (0.04; −0.153 to 0.016)		−.10		.11							
	Implementation leadership characteristics	0.54 (0.17; 0.195 to 0.879)		.63		.002							
	Implementation leadership × age	−0.01 (0.00; −0.017 to −0.001)		−.53		.03							

^a^0=man, 1=woman.

^b^0=registered nurse diploma or bachelor of nursing degree, 1=nursing graduate degree or other advanced education.

^c^*F*_10,228_=15.18; *P*<.001.

## Discussion

### Overview

This was a cross-sectional exploratory correlational study that was conducted to examine the effects of implementation leadership characteristics of first-level leaders, technology characteristics, and nurses’ individual characteristics on nurses’ intention to use and actual use of mHealth applications in clinical practice. To date, no other studies could be identified that have examined the role of implementation leadership in relation to the intention to use and actual use of mHealth applications in nursing, highlighting the novelty of this study.

There were a number of key findings with respect to intention to use and actual use of mHealth applications among nurses. Greater perceptions of the usefulness of mHealth applications were associated with greater intention to use and actual use of mHealth applications by nurses. Higher perceptions of implementation leadership characteristics were associated with greater intention to use mHealth applications among nurses, with greater influence of implementation leadership among nurses with RN diplomas or bachelor of nursing degrees than among those with graduate degrees or other advanced education. Implementation leadership also influenced nurses’ actual use of mHealth applications, with implementation leadership characteristics having a greater effect on nurses aged 29 to 49 years than on those aged ≥50 years. Finally, voluntariness was found to not moderate implementation leadership. These findings are discussed in detail in the following subsections.

### The Effects of Perceived Usefulness and Perceived Ease of Use

Perceived usefulness was found to be the strongest predictor of both nurses’ intention to use and actual use of mHealth applications to support direct patient care. For intention to use, perceived ease of use was additionally found to be a significant predictor. The significant effects of perceived usefulness and perceived ease of use on nurses’ intention to use mHealth applications were expected and in line with results that have been found elsewhere [52,73-75], with perceived usefulness being the strongest predictor of intention to use [9,76]. The large effect size for the addition of perceived usefulness and perceived ease of use suggests that these variables are of practical importance in influencing nurses’ intention to use mHealth applications in their practice [77]. These findings concur with those in the systematic review by Gagnon et al [5] that found a similar significant importance of these variables on the use of mHealth applications, particularly of perceived usefulness. As such, these results provide support for the importance of evaluating nurses’ assessments of the potential contributions and impacts to nurses’ provision of direct patient care and workflows of any potential mHealth technologies by organizations before their deployment. Indeed, this provides further support for the need to involve nurses in early or all stages of the system life cycle [78] and the importance of person-centered design [79-81]. The moderate effect size of the addition of perceived usefulness provided support for the importance of assessing nurses’ perceptions of perceived usefulness as a necessary part of the planning and implementation of mHealth applications, reflecting the findings of the importance of this variable in shaping the actual use of these technologies in other studies [5].

It was notable that perceived ease of use was a significant predictor of nurses’ intention to use mHealth applications but not a significant predictor of nurses’ actual use of mHealth applications. These findings are similar to the results of the study by Maillet et al [82], who examined nurses’ use of electronic patient records and found perceived usefulness (captured by the concept of performance expectancy [3]) to have a positive and significant influence on nurses’ actual use of electronic patient records, whereas perceived ease of use (captured by the concept of effort expectancy [3]) did not to have a significant influence. The authors also found that the link between perceived ease of use and facilitating conditions (which captures some aspects of implementation leadership characteristics) were among the strongest relationships identified [49,82]. Possible explanations for the different relationships between perceived ease of use and intention to use and between perceived ease of use and actual use may relate to discrepancies between behavioral intentions and actual behaviors or the how actual use behaviors are reported [9,83-88] (greater valuing of the usefulness of the mHealth tool may override the perceptions of ease of use [82]).

### The Effects of Implementation Leadership on Actual Use of mHealth Applications

Implementation leadership had a strong significant direct effect on actual use of mHealth applications, which suggests that the skills of the person responsible for overseeing the use of mHealth applications are an important consideration when implementing mHealth applications for nurses providing direct patient care. On examination of the interaction variables, we found that age moderated the effects of implementation leadership on nurses’ actual use of mHealth applications, where implementation leadership had a greater influence on increasing actual use of mHealth applications among younger nurses than among older nurses. One possible explanation for this finding may relate to the degree of expertise and self-efficacy that develop with increasing age and experience [89-91]. Older nurses may be more likely to have established ways of learning and acculturating to changes in their own practice. As such, they may be less influenced by the implementation leadership behaviors of first-level leaders who are promoting the use of mHealth applications. Another possible explanation for the greater influence of implementation leadership among younger nurses may be that the implementation leadership behaviors of first-level leaders are insufficient to mitigate the barriers that older nurses face in using mHealth applications in practice. Several studies have found that older nurses were more reluctant, less comfortable, and less likely to use HIT [92-94], and it is possible that first-level leaders’ implementation leadership characteristics may be perceived as insufficient to support mHealth applications use by this group.

Finally, differences in the measurement of actual use may account for the contradictory findings in this study in comparison with the study by Venkatesh et al [51]. Venkatesh et al [51] used variety and frequency as a measure of actual use, whereas this study used the measure of actual use developed by Doll and Torkzadeh [48], which captures actual use as a multidimensional concept. The use of the measure of actual use developed by Doll and Torkzadeh [48] brings the focus into technology use from the perspective of providing value; Shachak et al [95] suggest that viewing use in the context of the value it adds allows for the linking of use behaviors to specific tasks. In comparison, measuring the frequency and type of use provides limited information and limits the interpretability of results; the question remains as to whether a high frequency of use translates into meaningful use or perhaps reflects challenges in use, which results in a greater amount of time spent using the technology.

Overall, the number of potential explanations for the moderating effect of age on implementation leadership spans a broad range of possibilities, which suggests that there remains a lack of clarity and underdevelopment related to the understanding of the role of age in influencing nurses’ actual use of mHealth applications in practice. Similar to the study by Guo et al [96] that found seemingly contradictory effects of personalization and privacy in influencing the use of technologies that varied by age groups, it is likely that there are additional factors influencing the interaction between implementation leadership and age, and the subsequent effects on actual use warrant further exploration.

### The Effects of Implementation Leadership on Intention to Use mHealth Applications

Implementation leadership had a weak, nonsignificant relationship with intention to use mHealth applications. However, testing for the effects of interaction variables revealed that education had a significant moderating effect on the relationship between implementation leadership characteristics and nurses’ intention to use mHealth applications. This result suggests that implementation leadership characteristics were more influential in predicting nurses’ intention to use mHealth applications among nurses with an RN diploma or a bachelor of nursing degree than among those with a nursing graduate degree or other advanced education. A possible explanation is the difference in the levels of autonomy in roles occupied by nurses with advanced degrees [97], thus attenuating the effects of implementation leadership on their use of mHealth applications. However, the proportion of participants with an RN diploma or a bachelor of nursing degree (259/288, 89.9%) compared with participants with a nursing graduate degree or other advanced education (29/288, 10.1%) represents a wide difference. In addition to the small effect size, cautious interpretation is required to understand the potential role of the interaction between nurses’ level of education and implementation leadership on nurses’ intention to use mHealth applications. Although we believe that it remains worth considering that implementation leadership behaviors may need to be tailored to support the different subgroups of nurses rather than taking a *one-size-fits-all* approach to the implementation of mHealth applications, further analyses of the relationships between the dimensions of implementation leadership and level of education are needed to better understand the nature and magnitude of the relationships.

### The Effects of Voluntariness on the Use of mHealth Applications

Although it was hypothesized that voluntariness would moderate the effect of implementation leadership characteristics on the use of mHealth applications, it was found that voluntariness did not moderate the effect of implementation leadership characteristics on either intention to use or actual use of mHealth applications. However, standing alone, voluntariness was a significant negative predictor of both intention to use and actual use of mHealth applications, as has been found in some studies [52], including among other health care professionals [98]. These results suggest that when the use of mHealth applications was optional, nurses had lower intention to use and actual use of mHealth applications. There is mixed support for the importance of voluntariness in predicting intention to use mHealth applications and technology [52,99].

With regard to considering the effects of voluntariness on intention to use, a small effect size was found for the addition of voluntariness [64]. This small effect size limits the interpretation of this finding in terms of practical implications [77]. As such, the small effect of voluntariness on intention to use mHealth applications may provide some reassurance when interpreting these results in the context of health systems where the use of HIT systems is typically mandatory and does not allow for voluntariness of use to be considered.

However, when considering nurses’ actual use of mHealth applications, a medium effect size was found for the addition of voluntariness, which suggests that there may be moderate practical implications when considering the effects of voluntariness on nurses’ actual use of mHealth applications [77]. The practical implications of this finding can be interpreted in different ways. One message that can be gleaned from this finding is that making the use of mHealth applications mandatory in health care settings, which reflects the reality of HIT implementation currently, is necessary to optimize nurses’ intention to use and actual use of mHealth applications. Indeed, this approach is the most common method used to conduct implementations of mHealth applications and HIT in health care systems. However, a challenge with this approach is the inability to understand the reasons behind why nurses elect to not adopt and use these *mandatory-to-use* technologies, which is the current status quo. Although individual-level characteristics undoubtedly play a role in shaping use behaviors, broader structural and contextual variables also play an important role.

Another important consideration is the overall inadequate understanding of the role of leadership in influencing technology use in mandatory settings. Indeed, there is little research on voluntary technology use (vs mandatory technology use) as related to HIT in health care systems; voluntariness is more typically examined in the context of enterprise systems in business [100]. One potential interpretation of the inverse relationships between voluntariness and the intention to use and actual use of mHealth applications is that, when given the option, nurses may choose to not use mHealth applications as a result of perceived insufficient support for the use of mHealth applications in practice or poorly designed technologies that do not support nursing practice and workflows [101-107]. Finally, it is important in the interpretation of these results to consider that although voluntariness reduced intention to use and actual use, actual use rates are low among all participants, regardless of whether the use of the technology is voluntary or mandatory.

### Limitations

There are some limitations to this study. The first limitation relates to sampling procedures and the resulting composition of the study sample. Respondents were restricted to English-speaking participants, largely excluding French-speaking nurses in Canada. Another limitation is that the breakdown of respondents by province in this study is not representative of the broader Canadian nursing workforce. This was due to the large variability in recruitment success resulting from varied processes for research participant recruitment via RN registration bodies across Canada (Multimedia Appendix 3 [45]).

Another limitation relates to some of the study measures used. First, although the study focus is on first-level leaders, the wording used in the ILS scale is “mHealth applications leader.” It is possible that the individuals that nurses view as being the “mHealth applications leader” may not always correspond to a first-level leader as we defined it in this paper, which has implications for nurses’ ratings on the ILS. Next, we attempted to mitigate the limitations of the intention-to-use measure by adding a validated instrument to capture actual use. Nevertheless, collecting the system logs of actual use of mHealth applications would provide more accurate measures of the frequency and nature of the use of mHealth applications by nurses and provide additional insight into the potential meaningfulness of each measure of use of mHealth applications; for example, understanding the purpose for the use of mHealth applications as indicated by self-reports can provide insight into whether greater amounts of time spent using the system is a meaningful indicator of the successful use of mHealth applications or whether it shows problems with the mHealth applications. Finally, the use of a web-based survey is accompanied by some limitations. The web-based recruitment and survey approach with nonprobability sampling did not make it possible to estimate response rates and limited the ability to make explicit plans for mitigating low response rates. As such, the limitations of the sampling frame in terms of the ability to represent the national nursing population were anticipated. A related limitation of this study was the inability to pursue the means of recruitment beyond the web-based survey, given budgetary and time constraints. Although low response rates were observed among studies conducted in the 2000s, and the suggestion was made that there would be a limited increase in effectiveness in web-based surveys of health care providers in the future [108], more recent studies seem to suggest otherwise; for example, it has been shown that web surveys can achieve high numbers of responses in relatively short periods of time [105,109,110]. Furthermore, other studies that have compared web-based versus paper-and-pencil survey methods provide support for the general equivalency of the response rates that can be achieved with either method of data collection [111,112], as well as other comparable features such as potential for other types of biases [113].

### Conclusions

Research in the realm of implementation leadership is moving beyond studying the presence or absence of leadership to studying which specific leadership behaviors are most important and for whom these leadership behaviors are important. A recent review of the concept of implementation leadership characteristics suggests that the concept continues to evolve; nevertheless, it holds potential promise for use in the context of nursing [114]. Along the same lines, a systematic review conducted by Gifford et al [115] focused on managerial leadership and sought to identify leadership behaviors that were associated with supporting research use among nurses. The findings from the review identified a range of leadership behaviors that included being change oriented, task oriented, and relation oriented, as well as being supportive and demonstrating commitment to research-based practices—behaviors that hold parallels with the dimensions of implementation leadership. The results from this study provide support for the attenuated effects of implementation leadership on both intention to use and actual use of mHealth applications in nursing and contribute to the body of work that aims to better understand and delineate what effective leadership behaviors to support the use of mHealth applications in nursing might look like.

Future research can build on the insights from this study by using qualitative approaches to develop deeper understandings of whether the functions and features of mHealth applications match with nurses’ cognitive and information flows, nurses’ workflows, and support for patient-centered care. Although this study provides detail regarding the nature of leadership in relation to mHealth implementation in nursing, further delineation of the concept of implementation leadership should be explored. In particular, a qualitative exploration of nurses’ knowledge of the titles, roles, responsibilities, available resources, and constraints of the first-level leaders responsible for the implementation and ongoing use of mHealth in health care systems may provide important contextual information to aid in interpreting the relationships found between implementation leadership characteristics and nurses’ mHealth use. Several other research directions can be explored related to the actual use of mHealth, including examining the relationships in the various dimensions of actual use, relationships between types and functionalities of mHealth and use, and relationships between intention to use and the dimensions of actual use, all of which can contribute to a more nuanced understanding of what a meaningful measure of actual use might be in the context of nursing.

## Appendix

Multimedia Appendix 1 Instrument psychometrics.

Multimedia Appendix 2 Variable details.

Multimedia Appendix 3 Recruitment details.

## Abbreviations

CHERRIES: Checklist for Reporting Results of Internet E-Surveys
HIT: health information technology
ILS: Implementation Leadership Scale
mHealth: mobile health
Nurse LEAD-IT mHealth: Nurse Leadership for Implementing Technologies-Mobile Health
RN: registered nurse
RQ: research question
TAM3: Technology Acceptance Model 3
UTAUT: Unified Theory of Acceptance and Use of Technology
