# Supplementary material for: Differing Effects of Implementation Leadership Characteristics on Nurses’ Use of mHealth Technologies in Clinical Practice: Cross-Sectional Survey Study
Source: JMIR Nurs. 2023 Aug 25;6:e44435. doi: 10.2196/44435 (PMC10492171; doi:10.2196/44435)
Supplement: Multimedia Appendix 1 [file nursing_v6i1e44435_app1.docx]

#### Multimedia Appendix of Supplementary Files

#### Appendix 1. Instrument Psychometrics

The survey included an adapted version of Doll and Torkzadeh’s Actual Use Scale [49, 50], Aarons et al.’s Implementation Leadership Scale [26], variables from Technology Acceptance Model 3 (TAM3) [9] and Unified Theory of Acceptance and Use of Technology [8], nurse demographic characteristics, and researcher developed questions on the nature of mHealth use in nursing (e.g., what functions of mHealth were used). All instruments were psychometrically evaluated. Principal component analyses (PCA) for all scales produced component solutions consistent with previous studies [6-7, 9, 52-56] with exception of the Doll and Torkzadeh’s Actual Use Scale adapted actual use scale.

For the actual use scale, a PCA with oblique oblimin rotation was conducted which extracted two components that explained 67.74% of the variance. The two extracted components centred around support for problem solving and decision-making (problem solving, decision rationalization) and work coordination and integration (horizontal and vertical integration). These findings do not reflect the 5-dimension structure of actual use as proposed in Doll and Torkzadeh’s original scale [49]. However, the two-component solution does reflect the findings of the scale as adapted and used among nurses by Maillet et al [50]. A possible explanation is that Doll and Torkzadeh’s [49] suggestioned that the nature of the sample may determine the number of factors of the actual use scale, as distinguishing between the dimensions of actual use may be more unclear among certain groups. In our study, it is possible that nurses did not distinguish between problem solving and decision rationalization dimensions as these processes are inextricably linked in nurses’ provision of direct patient care: one must be able to rationalize decisions made as a part of clinical problem solving. Similarly, nurses may not distinguish between horizontal integration, vertical integration, and patient care, as it can be argued that these processes all centre on providing comprehensive and continuous person-centered care where functioning as part of a multidisciplinary team is a core aspect of practice. A summary of the component structure statistics for all scales used in each group of variables (outcome, key predictors, control).

### Principal Components Analysis Matrices

#### Outcome Variables

Component Matrix of the Intention to Use Scale

| Item | Component 1 |
| --- | --- |
| BI: 2. Given that I had access to the mHealth, I predict that I would use it.. | .942 |
| BI: 1. Assuming I had access to the mHealth, I intend to use it. | .935 |
| BI: 3. I plan to use the mHealth in the next month | .765 |

Note. n=286; Eigenvalue for Component 1=2.348.

Pattern Matrix of the PCA for the 14-item Actual Use Scale

|  | Component | |
| --- | --- | --- |
|  | 1 | 2 |
| Problem solving: 1.  I use mHealth to decide how to best approach a healthcare problem. | 0.861 |  |
| Problem solving: 2.  I use mHealth to help me think through healthcare problems. | 0.928 |  |
| Problem solving: 3.  I use mHealth to check my clinical judgment against the data. | 0.849 |  |
| Decision rationalization: 4.  I use mHealth to help me explain my clinical decisions. | 0.870 |  |
| Decision rationalization: 5.  I use mHealth to control or shape the clinical decision process. | 0.884 |  |
| Decision rationalization: 6.  I use mHealth to improve the effectiveness and efficiency of the clinical decision process. | 0.805 |  |
| Horizontal integration: 7.  My nursing team and I use mHealth to coordinate our activities. |  | 0.889 |
| Horizontal integration: 8.  I use mHealth to coordinate care activities with nurses in my nursing team. |  | 0.918 |
| Horizontal integration: 9.  I use mHealth to exchange information with nurses in my nursing team. |  | 0.903 |
| Vertical integration: 10.  I use mHealth to keep my superiors informed of my care activities. |  | 0.798 |
| Vertical integration: 11.  I use mHealth to exchange information with people who report to me (orderlies, nursing associates, clerk, etc.). |  | 0.785 |
| Patient care: 12.  I use mHealth to deal more efficiently with the nursing care I provide to my patients. |  | 0.589 |
| Patient Care: 13.  I use mHealth to personalize more the care for my patients. | 0.446 | 0.457 |
| Patient care: 14.  I use mHealth to exchange information with patients. |  | 0.480 |

Structure Matrix of the PCA for the 14-item Actual Use Scale

|  | Component | |
| --- | --- | --- |
|  | 1 | 2 |
| Problem solving: 1.  I use mHealth to decide how to best approach a healthcare problem. | .85 |  |
| Problem solving: 2.  I use mHealth to help me think through healthcare problems. | .90 |  |
| Problem solving: 3.  I use mHealth to check my clinical judgment against the data. | .84 |  |
| Decision rationalization: 4.  I use mHealth to help me explain my clinical decisions. | .87 |  |
| Decision rationalization: 5.  I use mHealth to control or shape the clinical decision process. | .88 |  |
| Decision rationalization: 6.  I use mHealth to improve the effectiveness and efficiency of the clinical decision process. | .85 | .43 |
| Horizontal integration: 7.  My nursing team and I use mHealth to coordinate our activities. |  | .86 |
| Horizontal integration: 8.  I use mHealth to coordinate care activities with nurses in my nursing team. |  | .89 |
| Horizontal integration: 9.  I use mHealth to exchange information with nurses in my nursing team. |  | .88 |
| Vertical integration: 10.  I use mHealth to keep my superiors informed of my care activities. |  | .81 |
| Vertical integration: 11.  I use mHealth to exchange information with people who report to me (orderlies, nursing associates, clerk, etc.). |  | .76 |
| Patient care: 12.  I use mHealth to deal more efficiently with the nursing care I provide to my patients. | .55 | .72 |
| Patient Care: 13.  I use mHealth to personalize more the care for my patients. | .63 | .64 |
| Patient care: 14.  I use mHealth to exchange information with patients. |  | .51 |

#### Predictor Variables

Pattern Matrix of the PCA for the 14-item Implementation Leadership Scale

| Item | Component | | | |
| --- | --- | --- | --- | --- |
|  | 1 | 2 | 3 | 4 |
| ILS Knowledgeable leadership: 1.  The mHealth leader is knowledgeable about mHealth. |  | .84 |  |  |
| ILS Knowledgeable leadership: 2.  The mHealth leader is able to answer my questions about mHealth. |  | .87 |  |  |
| ILS Knowledgeable leadership: 3.  The mHealth leader knows what he or she is talking about when it comes to mHealth. |  | .93 |  |  |
| ILS Perseverant leadership: 1.  The mHealth leader perseveres through the ups and downs of implementing mHealth. |  |  |  | .83 |
| ILS Perseverant leadership: 2.  The mHealth leader carries on through the challenges of implementing mHealth. |  |  |  | .88 |
| ILS Perseverant leadership: 3.  The mHealth leader reacts to critical issues regarding the implementation of mHealth by openly and effectively addressing the problem(s). |  |  |  | .79 |
| ILS Proactive leadership: 1.  The mHealth leader has developed a plan to facilitate implementation of mHealth. |  |  | .74 |  |
| ILS Proactive leadership: 2.  The mHealth leader has removed obstacles to the implementation of mHealth. |  |  | .88 |  |
| ILS Proactive leadership: 3.  The mHealth leader has established clear department standards for the implementation of mHealth. |  |  | .85 |  |
| ILS Supportive leadership: 1.  The mHealth leader recognizes and appreciates employee efforts towards successful implementation of mHealth. | .91 |  |  |  |
| ILS Supportive leadership: 2.  The mHealth leader supports employee efforts to learn more about mHealth. | .89 |  |  |  |
| ILS Supportive leadership: 3.  The mHealth leader supports employee efforts to use mHealth. | .80 |  |  |  |

Note. n=287; Oblique promax rotation with Kaiser Normalization; Eigenvalue for Component 1=8.88, Eigenvalue for Component 2=.748, Eigenvalue for component 3=.571, Eigenvalue for component 4=.456).

Structure Matrix of the PCA for the 14-item Implementation Leadership Scale

| Item | Component | | | |
| --- | --- | --- | --- | --- |
|  | 1 | 2 | 3 | 4 |
| ILS Knowledgeable leadership: 1. The mHealth leader is knowledgeable about mHealth. | .689 | **.932** | .685 | .749 |
| ILS Knowledgeable leadership: 2. The mHealth leader is able to answer my questions about mHealth. | .700 | **.959** | .734 | .746 |
| ILS Knowledgeable leadership: 3. The mHealth leader knows what he or she is talking about when it comes to mHealth. | .685 | **.967** | .720 | .728 |
| ILS Perseverant leadership: 1. The mHealth leader perseveres through the ups and downs of implementing mHealth. | .785 | .736 | .681 | **.952** |
| ILS Perseverant leadership: 2. The mHealth leader carries on through the challenges of implementing mHealth. | .748 | .742 | .720 | **.962** |
| ILS Perseverant leadership: 3. The mHealth leader reacts to critical issues regarding the implementation of mHealth by openly and effectively addressing the problem(s). | .720 | .720 | .754 | **.925** |
| ILS Proactive leadership: 1. The mHealth leader has developed a plan to facilitate implementation of mHealth. | .628 | .763 | **.885** | .638 |
| ILS Proactive leadership: 2. The mHealth leader has removed obstacles to the implementation of mHealth. | .683 | .659 | **.921** | .671 |
| ILS Proactive leadership: 3. The mHealth leader has established clear department standards for the implementation of mHealth. | .624 | .679 | **.915** | .708 |
| ILS Supportive leadership: 1. The mHealth leader recognizes and appreciates employee efforts towards successful implementation of mHealth. | **.949** | .676 | .666 | .682 |
| ILS Supportive leadership: 2. The mHealth leader supports employee efforts to learn more about mHealth. | **.943** | .668 | .656 | .752 |
| ILS Supportive leadership: 3. The mHealth leader supports employee efforts to use mHealth. | **.927** | .700 | .657 | .792 |

Note. The highest loadings are bolded and correspond with the variable loadings in the pattern matrix.

Component Matrix of the Perceived Usefulness Scale

| Item | Component 1 |
| --- | --- |
| PU: 3. Using mHealth enhances my effectiveness in my job. | .935 |
| PU: 1. Using mHealth improves my performance in my job | .914 |
| PU: 4. I find mHealth to be useful in my job. | .914 |
| PU: 2. Using mHealth in my job increases my productivity. | .898 |

Note. n=286; Eigenvalue for Component 1=3.352.

Component Matrix of The Perceived Ease of Use Scale

| Item | Component 1 |
| --- | --- |
| PEOU: 3. I find mHealth to be easy to use. | .920 |
| PEOU: 4. I find it easy to get mHealth to do what I want it to do. | .874 |
| PEOU: 1. My interaction with mHealth is clear and understandable. | .787 |
| PEOU: 2. Interacting with mHealth does not require a lot of my mental effort. | .785 |

Note. n=286; Eigenvalue for Component 1=2.847

#### Control Variable

Component Matrix of the Voluntariness of Use Scale

| Item | Component 1 |
| --- | --- |
| Voluntariness: 2. My supervisor does not require me to use mHealth. | .950 |
| Voluntariness: 3. Although it might be helpful, using mHealth is certainly not compulsory in my job. | .928 |
| Voluntariness: 1. My use of mHealth is voluntary | .890 |

Note. n=286; Eigenvalue for Component 1=2.608
