# Supplementary material for: Differing Effects of Implementation Leadership Characteristics on Nurses’ Use of mHealth Technologies in Clinical Practice: Cross-Sectional Survey Study
Source: JMIR Nurs. 2023 Aug 25;6:e44435. doi: 10.2196/44435 (PMC10492171; doi:10.2196/44435)
Supplement: Multimedia Appendix 2 [file nursing_v6i1e44435_app2.docx]

#### Multimedia Appendix of Supplementary Files

### Appendix 2: Variables

### Outcome variables

Bivariate Correlation Matrix of Study Variables

| Variable | 1 | 2 | 3^a^ | 4^a^ | 5 | 6 | 7 | 8 | 9 | 10 |
| --- | --- | --- | --- | --- | --- | --- | --- | --- | --- | --- |
| 1. Intention to use | - |  |  |  |  |  |  |  |  |  |
| 2. Actual use | **.35**** | - |  |  |  |  |  |  |  |  |
| 3. Gender ^a^ | .02 | -.06 | - |  |  |  |  |  |  |  |
| 4. Education ^a^ | -.02 | -.02 | .03^b^ | - |  |  |  |  |  |  |
| 5. Age | -.05 | -.06 | .11 | **.13*** | - |  |  |  |  |  |
| 6. Previous experience - mHealth at work | -.08 | -.07 | .02 | -.03 | **.42**** | - |  |  |  |  |
| 7. Previous experience – non-work mobile | .01 | -.03 | .09 | .04 | **.26**** | **.26**** | - |  |  |  |
| 8. Voluntariness | **-.17**** | **-.30**** | **-.17**** | **.13*** | **-.26**** | -.07 | **-.16*** | - |  |  |
| 9. Perceived usefulness | **.58**** | **.45**** | -.10 | -.06 | -.10 | -.07 | -.05 | -.03 | - |  |
| 10. Perceived ease of use | **.51**** | **.20**** | -.02 | -.02 | **-.20**** | **-.14*** | .004 | .04 | .**56**** | - |
| 11. Implementation leadership characteristics | **.27**** | **.41**** | .01 | **-.16**** | **.17**** | .02 | **.13*** | **-.50**** | **.32**** | **.26**** |

*p < .05. **p < .01. ***p < .001.

Note. Gender (0= male, 1= female), Education (0 = RN diploma or Bachelor of Nursing Degree, 1= Nursing Graduate Degree or Other).

^a^Point biserial correlations (rpb) were computed for gender and education in relation to all variables except each other.

^b^Phi (φ) was computed for the relationship between gender and education. Pearson’s correlations (r) were computed for all other bivariate relationships.

### Outcome variables

The outcome variables were nurses’ intention to use mHealth and nurses’ actual use of mHealth. Intention to use refers to nurses' plan to use mHealth as part of their clinical practice. The intention to use a technology is often considered a precursor and/or proxy for actual technology use behaviours and considered as the latter, in this study. The measure for intention to use comprised three items adapted from Venkatesh and Bala’s TAM3 [9]. Respondents were instructed to indicate the extent of agreement with these statements on a 7-point Likert scale, from 1 (strongly disagree) to 7 (strongly agree), with higher scores indicating stronger intentions to use mHealth.

To mitigate the limitations of using only intention to use as the measure of nurses’ mHealth use, actual use was also captured. We used Doll and Torkzadeh’s [49] measure of system-use adapted by Maillet [50], which has been validated in the context of the Canadian healthcare system (Cronbach's α = 0.93). Maillet’s [50] measure of actual use is a 14-item scale with five sub-scales that refer to the specific purpose for using the technology for problem solving, decision rationalization, horizontal integration, vertical integration, and patient care. Each item is scored on a 5-point scale indicating the degree to which the respondent perceives each statement to apply to them, ranging from 0 (not at all) to 4 (a great deal). The sum of all items was computed to create the actual use total score, where higher scores indicate greater use of mHealth for specific purposes [9, 49-50].

### Key predictor variables

The key predictor variables in this study were implementation leadership characteristics, perceived usefulness, and perceived ease of use of technology. Implementation leadership characteristics were measured using the staff version of the Implementation Leadership Scale (ILS) [26]. The ILS asks respondents to reflect on the specific leadership behaviours of the “first-level” leader in charge of implementation of mHealth, recognizing their key positioning to facilitate implementation [26]. The ILS is a 12-item scale with four sub-scales that refer to specific leadership behaviour traits of first-level leaders: proactive leadership, knowledgeable leadership, supportive leadership, and perseverant leadership. There are three items per sub-scale. Each item is scored on a 5-point scale indicating the degree to which the leader in charge of implementation in the unit/department performs specific behaviour, ranging from 0 (not at all) to 4 (to a very great extent). A score for each subscale was computed from the mean of items for each of the scale dimensions. The mean of the subscale scores was then computed as the total ILS score [26].

Perceived usefulness refers to nurses’ perceptions of how useful mHealth is in their work. The measurement of perceived usefulness was adapted from Venkatesh and Bala’s TAM3 [9]. Items were adapted to specify mHealth as the type of technology used, as per with recommendations by Davis et al. [6]. Respondents were instructed to indicate the extent of agreement with the instrument items on a scale from 1 (a very small extent) to 7 (a very large extent). The perceived usefulness variable was measured with a set of four items, with higher scores indicating greater perceptions of usefulness. The variable was formed by taking the mean of the items.

Perceived ease of use refers to one’s perception of how it is to use the mHealth. The measure of perceived usefulness was adapted from Venkatesh and Bala’s TAM3 [9] and comprise a subset of 4 items from Davis et al.'s [6] early TAM studies. Like the measure of perceived usefulness, items were adapted to specify mHealth as the type of technology used [6]. Respondents were instructed to indicate the extent of agreement with the items on a scale from 1 (a very small extent) to 7 (a very large extent). Perceived ease of use was computed by taking the mean of the four items; higher scores indicate greater perceived ease of use.

### Control variables

Control variables included voluntariness (a technology characteristic), previous experience with technology (individual characteristic related to technology), and nurse demographic characteristics (age, gender, education).

Voluntariness refers to the degree to which the use of mHealth is a mandatory or a voluntary component of nurses’ jobs. Three items were used to measure voluntariness, drawn from Moore and Benbasat [51]. All items were measured on a 7-point Likert scale (1 = strongly disagree and 7 = strongly agree). The variable was formed by taking mean of the three items.

Previous experience with mobile technology was conceptualized in this study as the two forms of previous experience that have been identified in the literature: i) experience as exposure/familiarity to similar technologies and, ii) experience with the specific technology in question. Both forms of experience have been associated with technology use behaviours and found to moderate the effects of other variables. However, no studies could be identified that made the distinction between the different forms of experience, nor examined if there is a difference in effects between the two. Previous experience was conceptualized in this study similar to previous studies, with experience representing the passage of time from the initial use of the technology up to the present [8, 52]. Previous experience as experience with *similar technologies* was operationalized by asking for an estimate of the month and year that the nurse first used mobile devices outside of work. The total number of months since first use of mobile devices outside of work were computed and used in the analyses as done by Venkatesh et al. [9]. Previous experience in the form of experience with the *specific technology in question* was operationalized by asking for an estimate of the month and year that the nurse first had access to employer-provided mHealth for use in their current nursing job from which the total number of months was computed and used in the analyses [52].

Nurse demographic characteristics included age, gender, and education, reflecting individual characteristics identified in both the technology use literature and nurses’ research utilization literature. Age in years was calculated from the participant’s report of their year of birth and month of birth. While studies on nurses’ use of research found no association between age and nurses’ research use [35-36], age has been identified in the technology use literature as influencing individuals’ perceived ease of use, perceived usefulness, attitudes towards technologies [8-9]. Age has also been found to moderate the effects of key relationships in technology acceptance models [8]. Gender was collected by asking respondents to identify as male, female, prefer not to say, other. However, gender was dichotomized into the categories female and male for analyses given the small percentage (2.9%) of participants responding with “prefer not to say” and “other.”; responses from individuals within those categories were removed. Previous research has found that gender roles and norms influence attitudes toward and actual use of technologies [8, 53]. For instance, it has been found that men show more favourable beliefs about the value of technology use and exhibit greater self-confidence in the ability to effectively learn and use technology as compared to women [120]. Others have found enduring differences related to beliefs of the value of technology use by gender over the span of 20 years [120]. It has also been found that perceived usefulness has a greater influence among men on deciding whether or not to use a new technology, whereas among women, perceived ease of use was found to play a greater role [121]. Gender roles have also been found to moderate the relationships between perceived usefulness and intention to use, and perceived ease of use and intention to use [8, 53]. It has also been suggested that gender and age appear to work in combination, although the interplay between age and gender is poorly understood [8]. Education was collected by asking participants to indicate the highest type of nursing degree that the individual had completed (RN diploma, Bachelor of Nursing, Master of Nursing, or PhD). For analyses, nursing education was dichotomized into two groups: i) RN diploma/ Bachelor of Nursing group and ii) Nursing Graduate Degree/Other group. This grouping was based on findings from the research utilization literature where having a graduate degree has been associated with increased research utilization when compared to diploma and bachelor of nursing degrees, and finding no differences when comparing research utilization between bachelor of nursing and diploma degrees [36].
