# Supplementary material for: Differing Effects of Implementation Leadership Characteristics on Nurses’ Use of mHealth Technologies in Clinical Practice: Cross-Sectional Survey Study
Source: JMIR Nurs. 2023 Aug 25;6:e44435. doi: 10.2196/44435 (PMC10492171; doi:10.2196/44435)
Supplement: Multimedia Appendix 3 [file nursing_v6i1e44435_app3.docx]

#### Multimedia Appendix of Supplementary Files

#### Appendix 3. Recruitment details

The majority of responses were from Saskatchewan (44.4%, *n* = 128), followed by Alberta (21.9%, *n* = 63), likely reflecting the well-established and efficient processes for recruiting RN registrants for research participation in those provinces. Namely, both provincial RN registration bodies had mailing lists of potential RN research participants that could be made readily available to researchers upon receiving the appropriate administrative and ethical approvals. It is also worth noting that the particularly high response rates from Saskatchewan is likely reflective of two key ways that the regulatory body supported the recruitment efforts of this research study that reflect best practices in Web survey implementation [45]. First, the survey advertisement was sent directly from the regulatory body (versus the researcher) to recipients, providing legitimacy and trust in the contents of the email. Secondly, the Saskatchewan RN regulatory body provided recurring email reminders (2 follow-up emails) that were sent out to participants as a standard service provided by their research support office. In contrast, other regulatory bodies required additional fees for this service, resulting in the inability to pursue these reminders given the available resources. It is also notable that in Ontario, despite being the most populous province, we had the lowest number of respondents in this sample. This is largely because at the time of the study, the only way to reach RN registrants in Ontario was by physical mail, with no electronic communication option. Given the nature of the study focusing on technology use and the additional resources that would be required to send out paper copies of the survey, we believe we had limited ability to advertise and recruit to Ontario RNs.
